# CYR61 Expression Is Induced by IGF1 and Promotes the Proliferation of Prostate Cancer Cells Through the PI3/AKT Signaling Pathway

**DOI:** 10.3390/ijms26188991

**Published:** 2025-09-15

**Authors:** Greisha L. Ortiz-Hernández, Carmina Patrick, Stefan Hinz, Mark A. LaBarge, Yun R. Li, Susan L. Neuhausen

**Affiliations:** 1 Department of Population Sciences, Division of Biomarkers of Early Detection and Prevention, Beckman Research Institute of City of Hope, Duarte, CA 91010, USA; cpatrick@coh.org (C.P.); shinz@coh.org (S.H.); mlabarge@coh.org (M.A.L.); sneuhausen@coh.org (S.L.N.); 2 Department of Radiation Oncology, City of Hope Comprehensive Cancer Center, 1500 E Duarte Rd, Duarte, CA 91010, USA; yunroseli@coh.org

**Keywords:** CYR61, prostate cancer, IGF1, PI3/AKT, MAPK, metastasis, metabolism

## Abstract

Cysteine-rich angiogenic inducer 61 (CYR61) promotes prostate cancer (PCa) cell growth, but its role in disease progression remains unclear. Given its insulin-like growth factor (IGF)-binding domain and the known involvement of insulin-like growth factor-1 (IGF1) in PCa, we investigated the molecular interplay between CYR61 and IGF1. CYR61 was silenced using small interfering RNA (siRNA) in prostate carcinoma 3 (PC3), lymph node carcinoma of the prostate (LNCaP), and androgen receptor (AR)-positive 22Rv1 cells, followed by assessments of their proliferation, viability, colony formation, migration, and signaling pathway activation. CYR61 knockdown significantly reduced cell growth, viability, prostasphere formation, and migration across all three cell lines. Mechanistically, CYR61 silencing inhibited PI3K/AKT signaling but had no effect on MAPK activation. In addition, treatment with recombinant IGF1 induced CYR61 expression in a time-dependent manner, and the inhibition of PI3K/AKT signaling suppressed both CYR61 expression and cell proliferation. These findings suggest that IGF1 promotes PCa progression through CYR61 and that CYR61 may serve as a potential therapeutic target for limiting tumor growth and metastasis.

## 1. Introduction

Prostate cancer (PCa) remains a significant public health concern, ranking as the second leading cause of cancer-related mortality among men in the United States. Alarmingly, the incidence rate has risen by 3% every year since 2014, underscoring the urgent need for an improved understanding of the disease and its management [1]. PCa is a biologically heterogeneous disease influenced by a complex interplay of clinical and demographic risk factors, including genetic predisposition, race and ethnicity, obesity, and age. While localized PCa is often manageable with current therapeutic strategies, metastatic PCa presents a far more formidable clinical challenge, with a significantly poorer prognosis and limited treatment options [2]. Projections estimate that by 2030, nearly 192,500 men will be living with metastatic PCa, highlighting the critical need to elucidate the molecular mechanisms that drive disease progression and metastasis.

One protein of growing interest in cancer biology is cysteine-rich angiogenic inducer 61 (CYR61), also known as CCN1 [3,4]. CYR61 is a matricellular protein that plays multifaceted roles in regulating cell adhesion, migration, proliferation, differentiation, and apoptosis [5,6,7,8,9,10]. As a secreted component of the extracellular matrix, CYR61 interacts with integrins and heparan sulfate proteoglycans, influencing various signaling pathways involved in tumorigenesis. Despite its established roles in other malignancies, the specific function of CYR61 in PCa remains poorly understood. Notably, CYR61 contains an insulin-like growth factor-binding protein (IGFBP) domain, suggesting a potential regulatory relationship with insulin-like growth factor-1 (IGF1), a hormone known to promote mitogenic and anti-apoptotic effects in prostate epithelial cells [11].

IGF1 has been implicated in the initiation and progression of PCa, with elevated circulating levels associated with resistance to various chemotherapies [12]. Moreover, IGF1 signaling has been linked to the development of castration-resistant prostate cancer (CRPC), a lethal form of the disease characterized by continued androgen receptor (AR) activity despite androgen deprivation therapy [13]. Reduced expression of the IGF1 receptor (IGF1-R) has also been proposed as a biomarker for metastatic potential, further emphasizing the clinical relevance of this pathway [14]. However, the potential molecular interplay between CYR61 and IGF1 in PCa has not been systematically investigated. Therefore, elucidating whether CYR61 modulates or is modulated by IGF1 signaling could provide important insights into mechanisms driving metastasis and therapeutic resistance.

While not previously described in PCa, the relationship and potential targeting of the IGF1-CYR61 axis in metastatic osteosarcoma have been reported. Specifically, CYR61 triggers primary tumor vascularization via an IGF1-R-dependent epithelial to mesenchymal transition (EMT)-like process [14]. Given that approximately 70% of metastatic PCa patients harbor bone metastases [2], these findings raise the possibility that similar molecular interactions may be at play in PCa metastasis. Thus, investigating the IGF1-CYR61 relationship in PCa is both timely and warranted.

In this study, we explored the role of CYR61 and its potential crosstalk with IGF1 in metastatic PCa using well-established cell line models, including PC3 and LNCaP, and AR-positive 22rv1. We further examine the impact of CYR61 silencing on the aggressive properties of these cells in PCa. Our findings provide new insights into the molecular underpinnings of PCa progression and suggest that targeting the CYR61-IGF1 axis may represent a novel therapeutic strategy for combating metastatic disease.

## 2. Results

### 2.1. CYR61 Promoted PCa Cell Lines’ Viability and Proliferation

**Figure 1:**
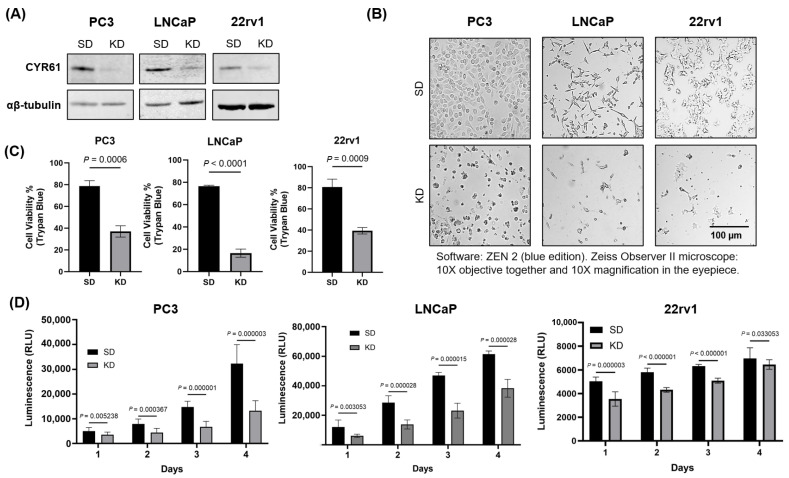
CYR61 knockdown produced morphological changes, decreased viability, and inhibited the proliferation of PCa cell lines. (**A**) The silencing efficiency of targeting CYR61 in PC3, LNCaP, and 22rv1 cells was detected by Western blot analysis. The cells were transfected with si-CYR61 pool of three different siRNA duplexes. (**B**) PC3, LNCaP, and 22rv1 cell morphology was visualized with the Zeiss Observer II. (**C**) PC3, LNCaP, and 22rv1 cell viability was detected by Trypan blue. (**D**) PC3, LNCaP, and 22rv1 cells were transfected with either si-CYR61 (KD) or si-Scramble Duplex (SD) for up to 4 d, and cell metabolic activity was evaluated by Cell Titer-Glo^®^ cell viability assay. Error bars represent mean ± standard deviation from a minimum of three technical replicates. Two-sample comparisons were determined using the two-tailed Student *t*-test. For multiple comparisons, two-way ANOVA was employed. *p* values < 0.05 were considered statistically significant.

We investigated the effects of CYR61 on the biological functions of PCa cell lines PC3, LNCaP, and 22rv1. A pool of three different CYR61 siRNA duplexes was used to transfect the cell lines. Transient silencing of CYR61 in PC3, LNCaP, and 22rv1 cells led to the robust depletion of the protein compared to cells transfected with the SD control (Figure 1A), as validated by Western blotting. CYR61 KD cell lines showed increased cell death compared to SD cells that had a relatively normal morphology with fewer floating cells and features of cell death (Figure 1B). The KD of CYR61 with CYR61 siRNA oligo reduced the viability of PC3 cells by 41.7% (*p* = 0.0006) after 72 h compared to the SD control. LNCaP cells showed an even larger decrease in cell viability with a decrease of 60.03% (*p* < 0.001) compared to the SD control. In addition, 22rv1 KD cells showed a 41.4% (*p* = 0.0009) decrease in cell viability compared to the SD group (Figure 1C).

To determine if there were differences in the metabolic activity of PC3, LNCaP, and 22rv1 cells when CYR61 was silenced, we used the CellTiter-Glo^®^ 2.0 assay to evaluate the effect of CYR61 KD on cell proliferation by quantifying ATP, which indicates the presence of metabolically active cells. AR is expressed in our highly proliferative (passage 9) LNCaP and 22rv1 cell lines, whereas PC3 has relatively low basal levels of AR and is unresponsive to androgen stimulation. CYR61 KD significantly inhibited the proliferation of PC3, LNCaP, and 22rv1 cells in a time-dependent manner, regardless of their AR sensitivity (Figure 1D). These results demonstrated that CYR61 KD affected cancer growth in the highly invasive PC3, androgen-sensitive LNCaP, and partially androgen-sensitive 22rv1 cell lines.

### 2.2. Silencing of CYR61 Inhibited Prostasphere Formation, Clonogenicity, Wound Healing, and Migration of PCa Cell Lines

**Figure 2:**
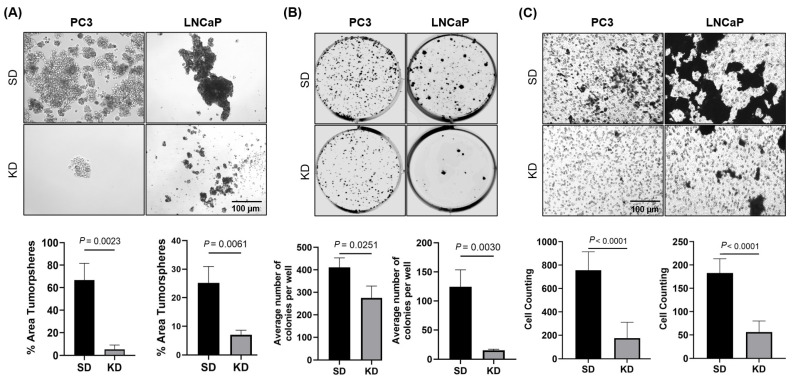
CYR61 knockdown repressed prostasphere formation, clonogenicity, and invasion of PCa cells. (**A**) Individual siRNA-mediated knockdown of CYR61 compared to SD control prostaspheres visualized using the Zeiss Observer II microscope. The percentage of tumorsphere area was quantified (bottom graphs) from triplicate images per experiment using ImageJ software (version 1.54f). (**B**) Representative images of clonogenic assay plates showed a decrease in colony formation in PC3 and LNCaP cells with CYR61 knockdowns compared to SD control cells. (**C**) PC3 and LNCaP cell proliferation detected by the Transwell migration assay. Error bars represent mean ± standard deviation from a minimum of three technical replicates. Two-sample comparisons were determined using the two-tailed Student *t*-test. For multiple comparisons, two-way ANOVA was employed. *p* values < 0.05 were considered statistically significant.

Using the prostasphere assay, we investigated the impact of the reduction in CYR61 on the stemness properties of PC3 and LNCaP. As depicted in Figure 2A, PC3-KD (*p* = 0.0023) and LNCaP-KD (*p* = 0.0061) cells exhibited a smaller number and size of prostaspheres compared to the PC3-SD and LNCaP-SD cells. These findings suggest that the downregulation of CYR61 diminished the ability of PC3 and LNCaP cells to grow as non-adherent prostaspheres.

The aggressiveness of cancer cell lines is associated with their ability to grow independently as colonies in vitro. Assessing colony formation is the standard method for measuring both proliferation and the ability of cancer cells to colonize and survive, serving as indicators of metastatic potential. When CYR61-expressing PC3, LNCaP, and 22rv1 cells were plated, they exhibited a notably high colony count (~120–450) (Figure 2B and Supplementary Figure S1). Conversely, the KD of CYR61 significantly diminished the clonogenic capacity of PC3 clones (*p* = 0.0251) and rendered LNCaP (*p* = 0.0030) and 22rv1 (*p* < 0.001) clones nearly incapable of growth.

To determine the extent to which intracellularly suppressed CYR61 regulates PCa cell migration, we transiently reduced CYR61 protein expression levels (see verification of CYR61 suppression; Figure 1A) in PC3 and LNCaP cell lines and analyzed cell migration using a 2D Transwell co-culture migration assay. Reducing CYR61 resulted in a significant decrease (*p* < 0.001) in cell migration in PC3 and LNCaP cells compared to the control SD groups, as shown in stained cells on the lower surface of the membrane (Figure 2C).

**Figure 3:**
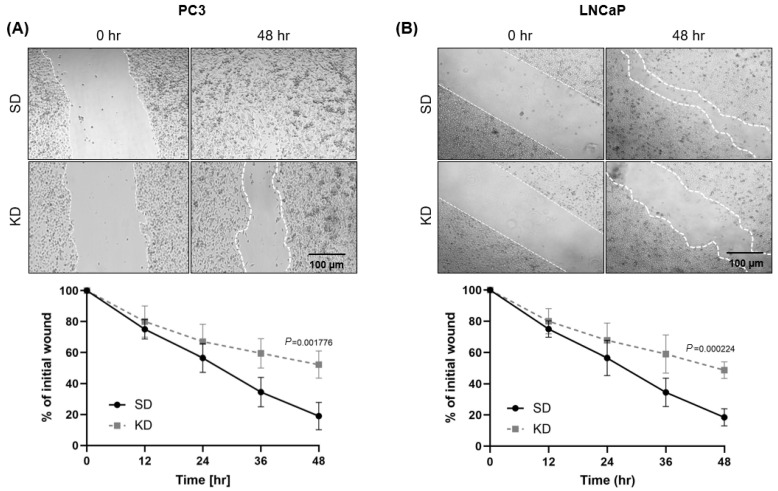
CYR61 knockdown repressed the migratory capacity of PCa cells. An individual siRNA-mediated knockdown of CYR61 compared to SD control visualized using the Zeiss Observer II microscope. (**A**) PC3 and (**B**) LNCaP cell migration detected by a wound-healing assay. Error bars represent mean ± standard deviation from a minimum of three technical replicates. Two-sample comparisons were determined using the two-tailed Student *t*-test. *p* values < 0.05 were considered statistically significant.

As CYR61 is known to promote metastasis in osteosarcoma and αv β3-mediated cell migration [15], we investigated whether depleting CYR61 affected the migratory ability of PC3 and LNCaP cells, measured by the relative wound closure area at 48 h. We found that PC3-SD cells efficiently closed the wound within 48 h, whereas PC3-KD cells exhibited significantly poorer wound closure (*p* = 0.001776) (Figure 3A). The LNCaP-SD cells exhibited a lower wound closure rate in comparison to PC3-SD cells, and the LNCaP-KD cells exhibited significantly less wound closure (*p* = 0.000224), compared to LNCaP-SD cells (Figure 3B).

### 2.3. Effect of CYR61 Silencing on the AKT, MAPK, and AR Signaling Pathways in PCa Cells

**Figure 4:**
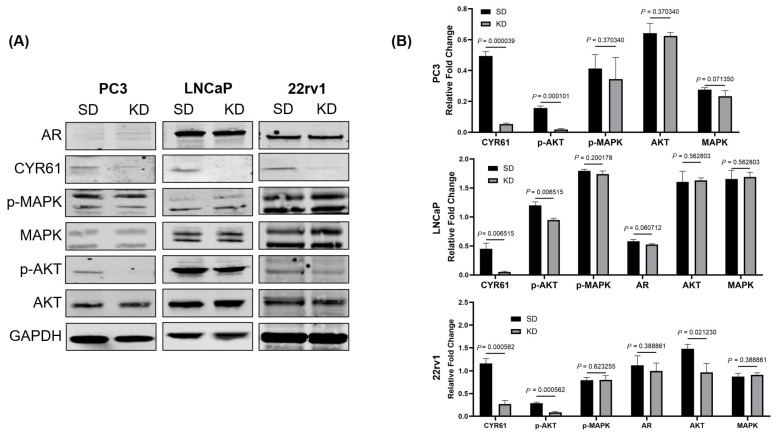
Effect of CYR61 knockdown on the AKT, MAPK, and AR signaling pathways. (**A**) Protein expression of AR, CYR61, p-MAPK, MAPK, p-AKT, and AKT in PC3, LNCaP, and 22rv1 cells transfected with either CYR61-KD or CYR61-SD control. Western blot images are representative of three technical replicates. (**B**) Data analysis and quantification of relative fold change. Error bars represent mean ± standard deviation from a minimum of three technical replicates. Multiple comparisons were determined using an unpaired *t*-test, and *p*-values < 0.05 were considered statistically significant.

Using a Western blot analysis, we measured the effects of CYR61 silencing on the AKT, MAPK, and AR signaling pathways. After silencing CYR61 (KD) in PC3, LNCaP, and 22rv1 cells, the levels of p-AKT in the three cell lines were significantly lower compared to the control SD group (Figure 4A,B). These results suggest that CYR61 silencing inhibits AKT phosphorylation in PC3, LNCaP, and 22rv1 cells. The molecular mechanism of CYR61 silencing in AR-V7 inhibition in PCa was further investigated in AR-positive LNCaP (highly proliferative) and 22rv1 cells (Figure 4A,B and Supplementary Figure S3). Compared with the control SD group, CYR61 KD cells showed significantly lower levels of AR-V7 in LNCaP cells (Figure 4A,B). CYR61 KD in LNCaP cells also led to a significant upregulation (*p* = 0.0049) of splicing factor 3B subunit 1 (SF3B1), whereas no such change was detected in PC3 or 22Rv1 cells (Supplementary Figure S4). This cell line-specific regulation suggests that CYR61 may influence splicing machinery in a context-dependent manner.

### 2.4. IGF1 Regulated CYR61 Expression Differently in PC3, LNCaP, and 22rv1 Cells

**Figure 5:**
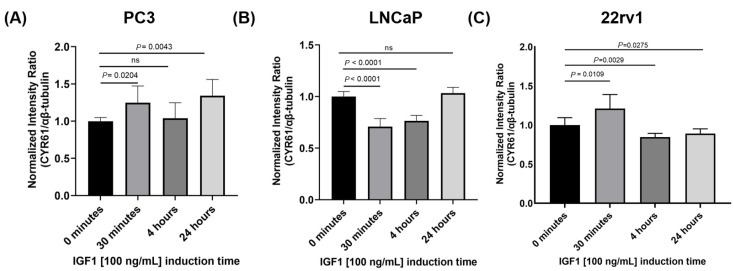
Baseline CYR61 expression levels varied in PC3, LNCaP, and 22rv1 cells in response to IGF1 induction. (**A**) PC3 cells exhibited an increase in CYR61 expression after IGF1 (100 ng/mL) treatment at 30 min and 24 h time points. (**B**) LNCaP cells induced with IGF1 (100 ng/mL) showed an inverse CYR61 expression, especially at 30 min and 4 h time points. (**C**) 22rv1 cells showed an increase in CYR61 at 30 min and a decrease at 4 and 24 h time points. Error bars represent mean ± standard deviation from a minimum of three technical replicates. For multiple comparisons, two-way ANOVA was employed. *p*-values < 0.05 were considered statistically significant.

PC3, LNCaP, and 22rv1 cells were induced with recombinant human IGF1 at different time points, and CYR61 expression was analyzed with the *In-Cell Western* (ICW) assay (Figure 5A–C). In PC3 cells (Figure 5A), recombinant human IGF1 treatment increased CYR61 expression at 30 min (*p* = 0.0204) and 24 h (*p* = 0.0043), but not at 4 h. In LNCaP cells (Figure 5B), IGF1 induction significantly decreased CYR61 expression at 30 min and 4 h post-induction (*p* < 0.001). However, CYR61 levels stabilized after 24 h, which suggests a feedback loop increases the levels of CYR61. In contrast, in 22rv1 cells (Figure 5C), IGF1 induced a significant increase in CYR61 at 30 min (*p* = 0.0109) and a significant decrease at 4 h (*p* = 0.0029) and 24 h (*p* = 0.0275) compared to the untreated (0 min) group.

### 2.5. IGF1 Induced Dynamic Changes in α5 and β1 Integrin Expression Across PCa Cells

**Figure 6:**
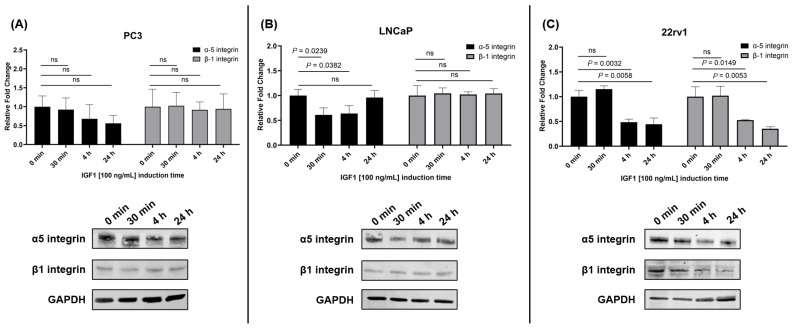
Expression dynamics of integrins α5 and β1 in IGF1-induced PCa cells. Protein expression of α5 and β1 integrins in (**A**) PC3, (**B**) LNCaP, and (**C**) 22rv1 cells after IGF1 (100 ng/mL) treatment at 30 min, 4h, and 24 h time points. Western blot images are representative of three technical replicates. Data analysis and quantification of relative fold change. Error bars represent mean ± standard deviation from three technical replicates. Multiple comparisons were determined using unpaired *t*-test, and *p*-values < 0.05 were considered statistically significant.

To determine if the IGF1-induced regulation of CYR61 expression is attributed to integrin dynamics, PC3, LNCaP, and 22rv1 (Figure 6) cells were treated with recombinant human IGF1 at different time points, and the protein expression levels of α5 and β1 integrin were measured. There were no significant differences in the expression of α5 and β1 in PC3 cells post-induction compared to the untreated group (Figure 6A). In LNCaP cells, induction with IGF1 for 30 min (*p* = 0.0239) or 4 h (*p* = 0.0382) significantly decreased the expression of α5 integrin compared to the untreated group. There was no significant difference for β1 integrin in these cells post-IGF1 induction (Figure 6B). In 22rv1 cells, there was a significant reduction for both α5 and β1 integrins at 4 h (*p* = 0.0149) and 24 h (*p* = 0.0053) post-induction with IGF1 (Figure 6C) compared to the untreated group.

### 2.6. IGF1 Induced Cell Proliferation via CYR61 Expression and the PI3K/AKT Signaling Pathway

To investigate the role of IGF1 in PCa proliferation and the regulation of CYR61 expression, we treated PC3, LNCaP, and 22Rv1 cell lines with IGF1 in the presence or absence of LY294002 (LY), a selective PI3K/AKT pathway inhibitor. Cell proliferation was assessed by measuring absorbance at 490 nm, while CYR61 protein levels were evaluated by ICW and normalized to β-tubulin.

**Figure 7:**
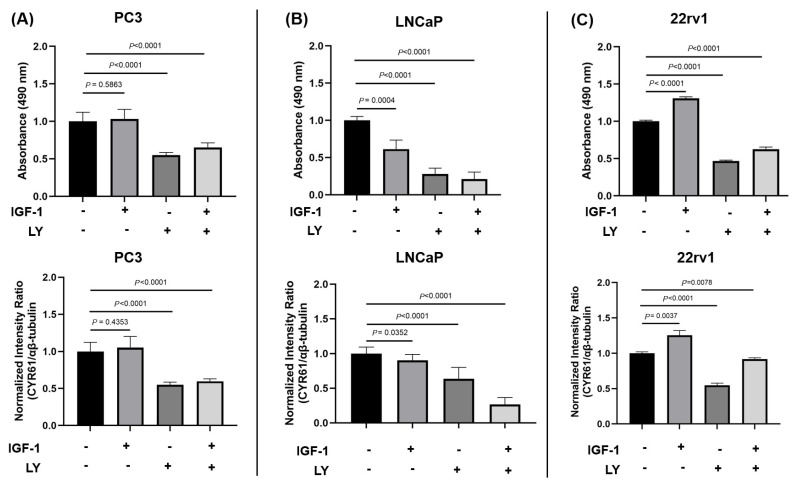
Effects of IGF-1 and LY induction on proliferation and CYR61 expression in PC3 (**A**), LNCaP (**B**), and 22rv1 (**C**) cells. The expression of CYR61 was assessed by ICW. The intensity ratio was normalized to αβ-tubulin expression. Results from proliferation (top) and expression (bottom) assays after 24 h IGF-1 (100 ng/mL) and LY (50 mM) treatment. Samples from both treatments were plated into 9 identical wells in a 96-well plate. All treatments were standardized relative to the untreated condition for each cell line. Error bars represent mean ± standard deviation from a minimum of three technical replicates. For multiple comparisons, two-way ANOVA was employed. The *p*-values were assessed relative to the untreated condition in each assay, with *p*-values < 0.05 considered statistically significant.

In PC3 cells (Figure 7A, top), IGF1 treatment alone did not significantly alter cell proliferation compared to the control (*p* = 0.5863). However, treatment with LY alone or in combination with IGF1 significantly reduced proliferation (*p* < 0.001), suggesting that basal PI3K/AKT activity supports proliferation in this androgen-independent line. Correspondingly, CYR61 protein expression (Figure 7A, bottom) was not significantly altered by IGF1 alone (*p* = 0.4353) but was significantly decreased upon LY treatment, either alone or in combination with IGF1 (*p* < 0.001).

In contrast, highly proliferative LNCaP cells (Figure 7B), which are androgen-sensitive, showed a significant decrease in proliferation upon IGF1 stimulation (*p* = 0.0004), an effect that was exacerbated by LY treatment (*p* < 0.001), indicating that IGF1 reduces proliferation via the PI3K/AKT pathway. Similarly, IGF1 significantly decreased CYR61 expression (*p* = 0.0352), and this reduction was enhanced by PI3/AKT inhibition, with LY alone or in combination significantly reducing CYR61 levels compared to IGF1 alone (*p* < 0.001).

22Rv1 cells (Figure 7C), which possess intermediate androgen responsiveness, showed the strongest proliferative response to IGF1 among the three lines (*p* < 0.001). This increase was significantly reversed by LY (*p* < 0.001), confirming the involvement of the PI3K/AKT pathway. CYR61 levels were also significantly elevated following IGF1 treatment (*p* = 0.0037), and this induction was partially inhibited by LY (*p* = 0.0078) in the presence of IGF1.

## 3. Discussion

The connective tissue growth factor, cysteine-rich protein, and nephroblastoma overexpressed gene (CCN) family are extracellular matrix proteins with reported roles in tumor invasion [16,17,18]. The CCN family of proteins is involved in different key molecular processes, including coordination in tumor microenvironments, tumorigenesis, and cancer metastasis [19,20,21]. In this paper, we focused on CYR61, also known as CCN1, which has been shown to act as a tumor-promoting factor and is likely a key regulator of cancer progression [22]. The IGF-binding protein domain of CYR61 is of particular interest because studies have reported that higher circulating levels of IGF1 are associated with metastatic disease and resistance to various chemotherapies [12]. We focused on characterizing the effect of CYR61 and its interplay with IGF1 on metastatic PCa and potential signaling pathway mechanisms. We studied three different PCa cell lines: (1) LNCaP cells, which express prostate-specific antigen (PSA) and partially express AR during high proliferation, and whose biological behavior is most similar to the majority of newly diagnosed PCa cases [23,24]; (2) PC3 cells, which do not express AR or PSA, are androgen-independent [24,25], and exhibit highly aggressive behavior, which is consistent with the biological behavior of advanced or treatment-resistant PCa; and (3) AR-positive 22rv1. With the use of these three models—LNCaP, PC3, and 22rv1—we aimed to represent a spectrum of PCa, encompassing both androgen-dependent and castration-resistant tumors. LNCaP cells, derived from lymph node metastasis, are androgen-sensitive and represent early-stage, hormone-responsive PCa. PC3 cells, originating from bone metastasis, are androgen-independent and highly aggressive, modeling advanced CRPC. 22Rv1 cells, derived from a relapsed xenograft, are androgen-independent but retain AR expression, making them a model for CRPC with active AR signaling. Our results demonstrated that CYR61 exerted pro-survival effects on PCa cells, and its expression was further induced with IGF1.

Previously, in cellular models of breast cancer, it was found that IGF1 promoted CYR61-induced cell growth and invasion [26], suggesting a regulatory relationship between IGF1 signaling and CYR61 expression. In our PCa models, we found that IGF1 stimulation led to a significant increase in CYR61 levels in PC3 and 22rv1 cells at the 30-min time point, and again in PC3 cells at 24 h. Conversely, in LNCaP cells, IGF1 induced a significant decrease in CYR61 levels at 30 min and 4 h, indicating a cell line-specific response to IGF1. Similar to what we observed in PC3 cells, Sarkissyan et al. [26] showed that in MCF7 breast cancer cells, CYR61 is upregulated significantly after 20 min of induction, with IGF1 demonstrating increased proliferation and invasion. A possible explanation for the observed decrease in CYR61 expression in LNCaP cells is that IGF1 stimulation alters the surface expression of specific integrins, such as α5 and β1, which are known to mediate CYR61 binding [27]. To explore whether these differences could be attributed to integrin-mediated dynamics, we examined the expression of α5 and β1 integrins following IGF1 treatment. In PC3 cells, no significant changes were observed in α5 or β1 integrin levels. This result suggests that the increase in CYR61 expression may be independent of these integrins or mediated through alternative integrin subtypes, such as αV, which has previously been shown to be upregulated in PC3 cells post-IGF1 stimulation [28]. In contrast, LNCaP cells exhibited a significant reduction in α5 integrin expression at 30 min and 4 h post-IGF1 treatment, with no significant change in β1 integrin levels. This reduction in α5 integrin may contribute to the observed decrease in CYR61 expression, as CYR61 primarily interacts with the cell surface via α5β1 integrin complexes [27]. These findings are consistent with prior reports indicating that IGF1 reduces αV integrin surface expression in LNCaP cells [28], further supporting the hypothesis that integrin downregulation may impair CYR61-mediated signaling in this cell line. In 22rv1 cells, IGF1 treatment resulted in a significant reduction in both α5 and β1 integrins at 4 h and 24 h. Despite this, CYR61 expression was significantly increased 30 min post-treatment, suggesting that early CYR61 induction may occur independently of α5β1 integrin regulation, potentially through transient signaling mechanisms or alternative integrin pathways. The later reduction in integrin expression may reflect a feedback mechanism or a shift in cellular adhesion dynamics.

Taken together, these results suggest that the IGF1-induced regulation of CYR61 is modulated by integrin expression in a cell line-specific manner. The differential expression of α5 and β1 integrins following IGF1 stimulation may influence CYR61’s availability and function, particularly in LNCaP cells, in which integrin downregulation correlates with reduced CYR61 levels. These findings underscore the complexity of IGF1-CYR61-integrin signaling and highlight the importance of cellular context in determining therapeutic responses in PCa. Consistently, high circulating levels of IGF1 have been positively associated with an increased risk of PCa [12,29]. In addition, AR has been shown to directly regulate the transcription of the IGF1 receptor in PCa cells [30]. A second possible explanation for the decrease in CYR61 in LNCaP cells is aberrant AR activation during high proliferation. The induction of these cells with recombinant human IGF1 and an increase in CYR61 expression can implicate a regulatory mechanism underlying AR expression through IGF1-mediated signaling pathways in PCa progression. Our findings suggest a potential novel interaction between AR and IGF1 signaling pathways that can contribute to PCa.

Together, our observations indicate for the first time that CYR61 expression can be induced by IGF1 in the context of PCa, showing that this protein is highly sensitive to growth factor induction. The upregulation of CYR61, which mediates cell proliferation, migration, and invasion, may result in increased metastasis. These findings are particularly significant for patients with elevated levels of circulating IGF1, who may face a higher risk of developing PCa and experience poorer survival outcomes. Interestingly, our results also demonstrate that CYR61 expression is rapidly upregulated in 22Rv1 PCa cells within 30 min of IGF1 stimulation, followed by a marked reduction at 4 h and 24 h post-treatment. This transient induction pattern suggests that CYR61 functions as an immediate early gene (IEG) in response to IGF1 signaling, a phenomenon commonly observed with growth factor-inducible genes that are rapidly but transiently activated to initiate downstream transcriptional programs [31]. The early spike in CYR61 may reflect the activation of the PI3K/AKT and MAPK/ERK pathways, both of which are downstream of the IGF1-R receptor and known to modulate CYR61 transcription [32,33]. The subsequent decline in CYR61 expression could be attributed to negative feedback regulation, receptor desensitization, or transcriptional repression mediated by downstream effectors, including FOXO transcription factors or other inhibitory regulators that restore homeostasis [34]. Additionally, CYR61 is known to be tightly regulated by temporal and context-specific cues, consistent with its dual role in promoting both proliferation and apoptosis depending on the cellular context [5]. This dynamic expression profile underscores the complexity of CYR61 regulation and highlights its potential role as a key mediator of IGF1-driven cellular responses in PCa.

A novel aspect of our study was the investigation of the ability of CYR61 to induce stemness. For that purpose, we optimized a prostasphere assay on CYR61 KD cells. This assay serves as the standard method for assessing the self-renewal capacity of cancer-stem-like cells (CSCs) in cell cultures [35]. In addition, this assay relies on the premise that only CSCs can thrive and proliferate without anchorage in the absence of serum. Aggressive cell lines, such as PC3, in which the expression of mesenchymal/stem cell molecular markers is exacerbated, CYR61 expression is elevated in comparison to other less-aggressive cell lines (Supplementary Figure S2). In cellular models of breast cancer, it has been suggested that mesenchymal-transformed non-invasive cells, such as MCF-7, show increased invasiveness and elevated CYR61 expression [36]. In our prostasphere models, we show for the first time that CYR61 KD impaired the formation of these spheres in PC3 and LNCaP cells. Similarly, in models of pancreatic carcinogenesis, CYR61 silencing reversed the EMT, blocked the expression of stem-cell-like traits, and inhibited migration [37].

We investigated whether the CYR61 upregulation due to IGF1 induction was mediated through the PI3K/AKT pathway, which is one of the central pathways in IGF1-induced PCa growth [38]. CYR61-KD PC3, LNCaP, and 22rv1 cells showed significant decreases in p-AKT levels, but not AKT, compared to CYR61-SD control cells. The MAPK pathway, which is the other key downstream pathway from IGF1, was also assessed [39]. CYR61-KD PC3, LNCaP, and 22rv1 cells did not show significant changes in levels of p-MAPK or MAPK. Together, these data suggest that IGF1-mediated CYR61 expression is intrinsically mediated through the PI3K/AKT signaling pathway and not the MAPK signaling pathway.

The AR signaling pathway regulates PCa cell proliferation and apoptosis [40]. An important mechanism through which PCa adapts to treatments targeting AR signaling is the constitutively active AR splice variants, especially AR-V7. The AR-V7 splice variant has been studied extensively, but its role in regulating the metastatic progression of castration-resistant PCa (CRPC) remains unclear [41]. We found that the KD of CYR61 in the highly proliferative LNCaP cells led to a significant reduction in AR-V7 expression, whereas in 22Rv1 cells, this effect was absent. This differential response likely reflects cell line-specific regulatory mechanisms and differences in AR signaling dynamics. LNCaP cells usually express only the full-length AR (AR-FL) and depend on ligand-activated AR signaling, making them highly sensitive to upstream regulators that affect AR gene transcription or mRNA splicing [42,43]. CYR61, a matricellular protein with known roles in cell signaling and gene transcription, may influence AR or AR splice variant expression through the modulation of integrin signaling or downstream effectors such as PI3K/AKT or MAPK pathways, which are known to intersect with AR signaling [5,44]. Conversely, 22Rv1 cells inherently express high levels of AR-V7 due to intragenic rearrangements and aberrant splicing, often independently of upstream signals [45]. This may render AR-V7 expression in 22Rv1 cells less susceptible to modulation by CYR61 KD, indicating that in this context, AR-V7 is constitutively expressed and uncoupled from extracellular matrix–mediated regulatory pathways. The reduction in AR-V7 in LNCaP cells suggests that alternative splicing is affected, possibly by changes in splicing factor availability [46]. Since CYR61 expression is regulated by hypoxia and undergoes alternative splicing itself in breast cancer [47], we hypothesized that CYR61 KD also could disrupt the balance of splicing regulation by shifting the splicing landscape away from producing variants such as AR-V7.

We assessed the expression of the SF3B1, a core component of the spliceosome, which is essential for the proper splicing of pre-mRNA and is implicated in cancer progression [48]. We observed that CYR61 KD in LNCaP cells led to a significant upregulation of SF3B1, whereas no such change was detected in PC3 or 22rv1 cells. This cell line-specific regulation suggests that CYR61 may influence splicing machinery in a context-dependent manner and might indicate a possible reprogramming of the splicing machinery, favoring more canonical splicing over alternative splicing, like AR-V7 production [49]. In contrast, 22Rv1 cells harbor intragenic rearrangements that drive constitutive AR-V7 expression, making them less responsive to upstream regulatory changes, including those mediated by CYR61. Similarly, PC3 cells lack AR expression altogether, which may explain the absence of SF3B1 modulation in response to CYR61 KD. These results highlight the contribution of AR variants and splicing factor dysregulation to tumor heterogeneity and the impact of therapeutic targeting of AR signaling in PCa. Future studies will be necessary to further elucidate the role of CYR61 in splicing regulation.

We found that IGF1 stimulated PCa cell proliferation and induced CYR61 expression through a PI3K/AKT-dependent mechanism, although the degree of response varied by cell line. The intermediate androgen-responsive 22Rv1 cells exhibited clear IGF1-dependent increases in proliferation and CYR61 expression, both of which were effectively blocked by PI3K inhibition. This suggests that IGF1 signaling through PI3K/AKT is critical in these contexts. Interestingly, in androgen-independent PC3 cells, IGF1 alone did not significantly impact proliferation or CYR61 expression, but LY treatment markedly reduced both, suggesting that constitutive PI3K/AKT activity may be maintaining basal levels of proliferation and CYR61 in the absence of exogenous IGF1. These findings highlight CYR61 as a downstream effector of PI3K/AKT signaling in PCa and suggest its potential as a biomarker or therapeutic target, particularly in tumors responsive to IGF1 signaling.

Our findings implicating IGF1 induction in CYR61 upregulation suggest important translational applications, particularly for understanding PCa risk across different populations [50]. Pooled analyses examining the associations of anthropometric, behavioral, and sociodemographic factors with circulating concentrations of IGF1 in 16,024 men from 22 studies report that Hispanic/Latino (H/L) men with PCa tend to have lower circulating levels of IGF1 and its binding proteins, which may reduce the amount of bioavailable IGF1. Despite this, high serum levels of IGF1 in some H/L men are still associated with increased PCa risk. In addition, low IGF1-R expression in this group was linked to a higher likelihood of metastatic disease [12]. Among African American men, specific genetic variants in IGF1 are associated with increased PCa risk [51]. Furthermore, circulating levels of IGF1 are known to be heritable, influenced by factors such as vitamin D, and have been linked to resistance to various chemotherapies [52,53]. These findings highlight how racial and ethnic differences in IGF signaling may contribute to disparities in PCa disease and progression. Consequently, increased IGF1-induced CYR61 activity, potentially acting through the classical PI3K/AKT signaling pathway, may represent a mechanism by which tumors evolve into more aggressive and invasive phenotypes. This signaling axis has been implicated in promoting EMT, extracellular matrix remodeling, and immune evasion in various cancers. Targeting CYR61 directly by using agents such as the monoclonal antibody, 093G9 has shown promise in preclinical models, in which it inhibited the proliferation, migration, and metastasis of breast cancer cells by downregulating AKT and ERK phosphorylation [54]. Moreover, neutralizing antibodies against CYR61 have demonstrated a significant suppression of estrogen-driven proliferation in MCF-7 cells [14]. Integrating CYR61 inhibition with existing therapies, such as IGF1R or PI3K inhibitors, may offer synergistic benefits by disrupting both upstream and downstream components of this oncogenic pathway, potentially overcoming resistance and enhancing therapeutic efficacy [26]. This multi-targeted approach could be particularly valuable in tumors exhibiting high CYR61 expression and aggressive phenotypes. Further studies are needed to validate these observations and to explore whether targeting CYR61 could serve as a therapeutic strategy for advanced PCa.

A limitation in our study is the use of three PCa cell lines. Although representative of androgen-dependent and castration-resistant tumors, the use of PC3, LNCaP, and 22rv1 cell lines may not reflect the full heterogeneity of PCa seen in patients and may not fully capture the complexity of PCa progression in vivo. Cell lines have inherent limitations, such as differences in genetic background and cellular behavior that may not accurately mimic the tumor microenvironment or the molecular interactions within a living organism. Therefore, while the findings provide valuable mechanistic insights, further research is needed to explore these additional pathways and validate the results in more complex and clinically relevant models. Future translational work will include mapping the expression of CYR61 and IGF1 across epithelial, stromal, and immune compartments of PCa-derived tissues using advanced single-nucleus RNA-seq technology. In addition, we will investigate associations of *CYR61* gene expression with genes and pathways upstream and downstream of CYR61 and PCa outcomes in a database of PCa tissue from diverse City of Hope patients. From the gene expression data of 491 PCa tissues in the Prostate Adenocarcinoma TCGA Firehose Legacy dataset, the expression levels for CYR61 varied over two-fold and depended on the patients’ tumor stage, location, and age at diagnosis. The existing literature supports the association of CYR61 expression with clinical outcomes in various cancers, including breast, hepatocellular, and PCa [9,55,56]. Our findings also suggest that integrin dynamics modulate the IGF1-induced regulation of CYR61 in a cell line-specific manner, which may reflect distinct tumor phenotypes and therapeutic vulnerabilities. Although we quantified α5 and β1 integrin expression post-IGF1 stimulation (Figure 6), we did not assess αV integrin or IGF1-R directly. Future studies incorporating flow cytometry or blocking experiments will be essential to fully elucidate the mechanistic role of integrin subtypes in CYR61 regulation and their potential as therapeutic targets. These insights may contribute to a better understanding of CYR61’s role in disease progression and its utility as a biomarker or intervention point in PCa treatment.

## 4. Materials and Methods

### 4.1. Cell Lines

PCa cell lines (PC3, LNCaP, and 22rv1) were obtained from the American Type Culture Collection (Manassas, VA, USA, Cat# CRL-1435, CRL-1740, and CRL-2505, respectively) and cultured in RPMI-1640 medium (Corning, Buffalo, NY, USA, Cat# 10-040-CM), supplemented with 10% (*v*/*v*) fetal bovine serum (FBS, Corning, Corning, Cat# MT35010CV), penicillin/streptomycin (Corning, Cat# 30-002-CI), and normocin 1G (Invivogen, San Diego, CA, USA, Cat# NC9390718). Cells were grown under 5% CO_2_ at 37 °C. Short tandem repeat service provided by ATCC (Cat# ATCC-135-XV) was used to authenticate the cell lines. Mycoplasma testing was conducted at least twice a year using the Lonza MycoAlertTM Mycoplasma Detection Kit (Lonza, Basel, Switzerland, Cat# LT07-218).

PC3 cells are androgen-independent and lack endogenous AR expression, representing an advanced, castration-resistant phenotype. In contrast, LNCaP cells are androgen-sensitive and express functional AR, making them a model for hormone-responsive PCa. 22Rv1 cells express both full-length AR and constitutively active AR splice variants, such as AR-V7, and are used to model castration-resistant PCa with partial AR signaling activity.

### 4.2. Antibodies

Rabbit antibodies targeting the following proteins were acquired from Cell Signaling Technology (Danvers, MA, USA): CYR61 (D4H5D) (Cat# 14479S), α/β-tubulin (Cat# 2148S), β-actin (13E5) (Cat# 4970), Phospho-AKT (Thr308) (Cat# 2965), Phospho-p44/42 MAPK (Erk1/2) (Cat# 9101S), Androgen Receptor (D6F11) (Cat# 5153), and Integrin α5 (Cat# 4705). In addition, we used a rabbit antibody targeting SF3B1 (ThermoFisher Scientific, Carlsbad, CA, USA, Cat# PA5-41723). Mouse monoclonal antibodies included CYR61 (A-10) (Santa Cruz Biotechnology, Dallas, TX, USA, Cat# sc-374129), Beta 1 (β1) Integrin (R&D Systems Inc., Minneapolis, MN, USA, Cat# MAB17781), and β1 Integrin (Developmental Studies Hybridoma Bank, Iowa City, IA, USA, Cat# P5D2).

### 4.3. Immunoblotting

Whole-cell lysates were prepared by lysing the cell pellet in 100 µL Invitrogen™ Cell Lysis Buffer II (Thermo Fisher Scientific, Waltham, MA, USA, Cat# FNN0021) on ice for 30 min, vortexing at 10 min intervals. The cell lysis buffer was supplemented with 1 mM Thermo Scientific™ PMSF Protease Inhibitor (Thermo Fisher Scientific, Cat# PI36978) and Halt™ Protease and Phosphatase Inhibitor Cocktail (Thermo Fisher Scientific, Cat# P178440). The cell extract was centrifuged at 13,000 rpm for 10 min at 4 °C. The supernatant was collected, and protein concentration of the lysates was determined using the Pierce™ BCA Protein Assay Kit (Thermo Fisher Scientific, Cat# 23225) to ensure equal loading of proteins separated on individual lanes by SDS-PAGE (NuPAGE 4–12%, Thermo Fisher Scientific, Waltham, MA, USA). Samples for immunoblot analysis were diluted in ultrapure water, Invitrogen™ 1× Bolt™ Sample Reducing Agent (Thermo Fisher Scientific, Cat# B0004), and Invitrogen™ NuPAGE™ Lithium Dodecyl Sulfate Sample Buffer (1×) (Thermo Fisher Scientific, Cat# NP0007) to a concentration of 50 µg of protein per 20 µL, then heated at 70 °C for 10 min. Electrophoresis was followed by protein transfer to polyvinyl difluoride membranes (Millipore Sigma, Burlington MA, USA, Cat# IPFL00010). Membranes were blocked for 1 h at room temperature with Intercept^®^ Blocking Buffer (Li-Cor, Lincoln, NE, USA, Cat# 927-60001) and probed overnight with appropriate primary antibodies. Membranes were then washed with TBS-Tween buffer (20 mM Tris-HCL, pH 7.6, 140 mM NaCl, and 0.2% Tween 20) three times for 5 min each. After washing, membranes were incubated with IRDye^®^ 680RD Goat anti-Rabbit IgG Secondary Antibody (Li-Cor, Cat# 926-68071) or IRDye^®^ 680RD Goat anti-Mouse IgG Secondary Antibody (Li-Cor, Cat# 926-68070). Washes were repeated after secondary antibody incubation. Membranes were imaged using a Li-Cor Odyssey scanner. Protein bands from at least 3 independent blots were scanned for each protein of interest, quantified using ImageJ software (National Institutes of Health, Bethesda, MD, USA, Fiji Version 1.44a), and normalized to α/β-tubulin or β-actin loading control protein bands to determine fold upregulation.

### 4.4. Cell Viability Assay

Trypan blue dye exclusion assay was employed to assess the effects of CYR61 small interfering (siRNA) on the viability of PC3, LNCaP, and 22rv1 cells. Following siRNA transfection, cells (both floating and adherent) were collected, pelleted by centrifuging at 1500 rpm for 5 min, and resuspended in 1 mL 1× phosphate-buffered saline (PBS). After pellet resuspension, 10 µL of cell suspension was combined with 10 µL Trypan blue (Thermo Fisher Scientific, Cat# MT25900CI), and live cells were counted using a Cellometer Vision CBA Image Cytometer (Nexcelom, San Diego, CA, USA). Cell viability was expressed as a percentage of the viability of the scrambled siRNA duplex (SD) control.

The CellTiter-Glo^®^ assay was used to assess cellular proliferation and viability. Following CYR61 siRNA transfection, PC3, LNCaP, and 22rv1 cells were seeded (5000 cells/well) in opaque-walled 96-well plates (Thermo Fisher Scientific, Cat# 12-566-620) and 100 µL complete media. Control wells containing medium without cells were used to determine background luminescence. Cells were assessed for 1 to 4 days post-transfection. After each time point, a volume of CellTiter-Glo^®^ 2.0 Reagent equal to the volume of cell culture medium present in each well (100 µL) was mixed for 2 min on an orbital shaker to induce cell lysis. Following this, the plate was incubated at room temperature for 10 min to stabilize the luminescent signal. The luminescence was recorded using an integration time of 0.25–1 s per well as per manufacturer’s instructions.

### 4.5. RNA Interference

PC3, LNCaP, and 22rv1 cells (50,000 cells per well) were cultured on 6-well plates and transfected after 24 h with either 50 nM or 100 nM siRNA for up to 96 h. The siRNA sequences used were as follows: si-CYR61 knockdown (KD, pool of three different siRNA duplexes from Santa Cruz Biotechnology, Cat# sc-39331). Cells were transfected using Interferin^®^ siRNA transfection reagent (Polyplus-transfection^®^, Illkirch, France, Cat# 409-01). SD (Dharmacon, Lafayette, CO, USA, Cat# D-001210-0105) was used as a non-targeting negative control. Protein depletion was assessed by immunoblotting.

### 4.6. Clonogenic Assay

PC3, LNCaP, and 22rv1 cells were transfected with CYR61 siRNA and grown in RPMI-1640 medium supplemented with 10% FBS for 72 h. Next, equal numbers of viable transfected cells were transferred to 6-well culture plates (500 cells/well for PC3 and 1000 cells/well for LNCaP and 22rv1). Colony formation plates were incubated for 10 days at 5% CO_2_ and 37 °C. Adherent colonies were washed with 1× PBS, fixed with 3:1 (*v*/*v*) methanol:acetic acid solution for 5 min, washed again with 1× PBS, stained with 0.5% crystal violet for 20 min, and then washed gently with tap water. Images of the stained colonies were acquired using a Li-Cor Odyssey scanner, and quantification was performed using the automated colony counting capability of ImageJ software following identical parameters for each well.

### 4.7. Prostasphere Formation Assay

Spheroid cultures from siRNA transfected cells were maintained using complete MammoCult™ medium (Stem Cell Technologies, Vancouver, BC, Canada, Cat# 05620) supplemented with hydrocortisone (0.48 g/mL, Millipore Sigma-Aldrich, Cat# H0135), heparin (4 g/mL Sigma-Aldrich, St. Louis, MO, USA, Cat# H3149), and 1% penicillin/streptomycin. PC3 and LNCaP cells were seeded at 50,000 cells per well and transfected with the various siRNAs. After 48 h, an equal number of viable cells (1000 cells/well) were harvested and resuspended 50 times in MammoCult™ medium to ensure a single-cell suspension. Cells then were seeded in untreated 24-well plates (Genesee Scientific, Morrisville, NC, USA, Cat# 25–102) with 0.5 mL MammoCult™ medium. Prostaspheres were grown for 10 d at 37 °C/5% CO_2_ and visualized in a Zeiss Observer II microscope (Zeiss, Jena, Germany). The prostasphere area was quantified from three independent images per individual treatment using ImageJ software.

### 4.8. Wound-Healing Assay

PC3 and LNCaP cells were examined for their mobility using a wound-healing assay. Following CYR61 siRNA transfection for 72 h, cells were collected and seeded into a 24-well plate (Thermo Fisher Scientific, Cat# 09-761-146) to reach semi-confluency. Confluent transfected cells were “scratch wounded” with a P200 pipette tip. Wound closure was monitored using a Nikon Eclipse Ti microscope. Over a 48 h time period, images were taken every 12 h and analyzed with ImageJ software. The average wound area relative to the initial wounding (0 h) was determined in three independent triplicate assays and compared to control cells transfected with SD negative control.

### 4.9. IGF1 Induction Treatment

To determine if IGF1 increased the expression of CYR61, PC3, LNCaP, and 22rv1 cells were induced with 100 ng/mL recombinant human IGF1 (Peprotech, Cranbury, NJ, USA, Cat# 100-11) for 30 min, 4 h, and 24 h. CYR61 protein expression levels were measured at different time points. Experimental cells were plated, and once 70% confluent, they were switched to serum-free media with 0.1% BSA for overnight starvation. Immediately after exposure to IGF1 for the designated time, the cells were collected for protein extraction or evaluated for expression.

### 4.10. ICW Assay

The ICW assay was performed using the Odyssey Imaging System (LI-COR Biosciences, Lincoln, NE, USA) according to the manufacturer’s instructions. PC3, LNCaP, and 22rv1 cells were grown in 96-well plates until they reached 60–70% confluency and then fixed with 4% paraformaldehyde at selected time intervals post-transfection or IGF1 induction for the ICW assay. Then, cells were permeabilized with 0.5% Triton X-100 for 15 min at room temperature and blocked with LI-COR Odyssey Blocking Solution (LI-COR Biosciences) for 30 min. The cells were incubated at 4 °C overnight with appropriate primary antibodies. After three washes with 1× PBS, cells were stained with secondary antibodies at room temperature for 2 h and washed twice again with 1x PBS. The plates were scanned with the Odyssey CLx Infrared Imaging System (LI-COR Biosciences), and the integrated fluorescence intensities representing the protein expression levels were acquired using the software provided with the imager station (Empiria Studio 2.3, LI-COR Biosciences). The relative amount of protein was obtained by normalization to endogenous β-actin or α/β-tubulin in all experiments.

### 4.11. Transwell Migration Assay

Cell migration was assessed using 24-well ThinCert™ (Greiner Bio One, Frickenhausen, Germany, Cat# 662638) permeable inserts containing PET capillary pore membranes with 8.36 mm inner diameter, 10.34 mm outer diameter, 16.22 mm height, and 8 μm pore size. The assay was carried out once the transfection time ended. PC3 and LNCaP cells were trypsinized, and 1 × 10^5^ live cells/mL were resuspended in serum-free RPMI medium. While the cell suspensions were prepared, 500 μL of the chemoattractant (RPMI supplemented with 10% FBS) was dispensed into each well of the 24-well ThinCert™ plates and incubated at 37 °C for 1 h. The ThinCert™ inserts were placed in the wells containing pre-warmed chemoattractant, and 1 × 10^5^ live cells/mL (200 μL from the cell suspension) were added to the insert. Subsequently, the plates were incubated at 37 °C for 48 h. The medium in the inserts was then removed, and the membranes were washed twice in PBS. The PC3 and LNCaP cells that remained in the upper part of the insert were removed carefully with a PBS-soaked cotton swab. The cells that migrated towards or invaded the lower part of the chamber were fixed with 4% formaldehyde and stained with 0.5% crystal violet. The fixed cells were visualized on a Zeiss Observer II microscope and photographed. The total number of cells that migrated or invaded was counted manually using ImageJ software.

### 4.12. PI3K/AKT Inhibitor Induction Treatments

A solution of 50 mM of LY294002 (LY) (Cell Signaling Technology, Danvers, MA, USA; Cat# 9901), which is a PI3/AKT inhibitor, was used to determine its specific role in response to IGF1-mediated CYR61 upregulation. Cells were pretreated with LY for 1 h prior to the 24 h IGF1 induction. The IGF1/LY-induced changes in CYR61 expression levels were assessed using an ICW assay. Cell proliferation was assessed using the CellTiter 96^®^ AQueous One Solution Cell Proliferation Assay kit (Promega, Madison, WI, USA) as per the manufacturer’s instructions.

### 4.13. Statistical Analysis

Data are expressed as mean ± SEM from a minimum of three independent experiments. Statistical analysis was performed with GraphPad Prism version 9 (GraphPad Software, San Diego, CA, USA). Two-sample comparisons were determined using the two-tailed Student *t*-test. For multiple groups, we used two-way ANOVA. *p* values < 0.05 were considered statistically significant.

## 5. Conclusions

Our results suggest a potential mechanism by which IGF1 stimulates the expression of CYR61. This induction occurs differentially across the PC3, LNCaP, and 22Rv1 PCa cell lines. The PI3K/AKT signaling pathway mediates this IGF1-induced CYR61 expression, contributing to the cell survival, migration, proliferation, cell metabolism, and self-renewal capacity of stem-like cell populations. In addition, this pathway is associated with levels of AR-V7, an AR splice variant linked to therapy resistance.

## Data Availability

The data generated in this study are available from the corresponding author: Greisha L. Ortiz-Hernandez (gortizhernandez@coh.org).

## Supplementary Materials

The following supporting information can be downloaded at: https://www.mdpi.com/article/10.3390/ijms26188991/s1.

## Abbreviations

The following abbreviations are used in this manuscript:

AR	Androgen receptor
CCN	Connective tissue growth factor, cysteine-rich protein and nephroblastoma overexpressed gene
CRPC	Castration-resistant prostate cancer
CSC	Cancer stem-like cell
CYR61	Cysteine-rich angiogenic inducer 61
H/L	Hispanic/Latino
ICW	In-cell Western
IGF1	Insulin-like growth factor-I
IGF1-R	Insulin-like growth factor-receptor
KD	Knockdown
LNCaP	Lymph node carcinoma of the prostate
MAPK	Mitogen-activated protein kinases
p-AKT	Phosphorylated protein kinase B
p-MAPK	Phosphorylated mitogen-activated protein kinases
PBS	Phosphate-buffered saline
PC3	Prostate carcinoma 3
PCa	Prostate cancer
PFA	Paraformaldehyde
PI3/AKT	Phosphatidylinositol 3-kinase/protein kinase B
PSA	Prostate-specific antigen
SD	Scramble siRNA duplex
siRNA	Small interference RNA
TBS	Tris buffer saline
