# Supplementary material for: CYR61 Expression Is Induced by IGF1 and Promotes the Proliferation of Prostate Cancer Cells Through the PI3/AKT Signaling Pathway
Source: Int J Mol Sci. 2025 Sep 15;26(18):8991. doi: 10.3390/ijms26188991 (PMC12470006; doi:10.3390/ijms26188991)

Supplementary Figures

Figure S1. Silencing of CYR61 inhibited clonogenicity of 22rv1 cells

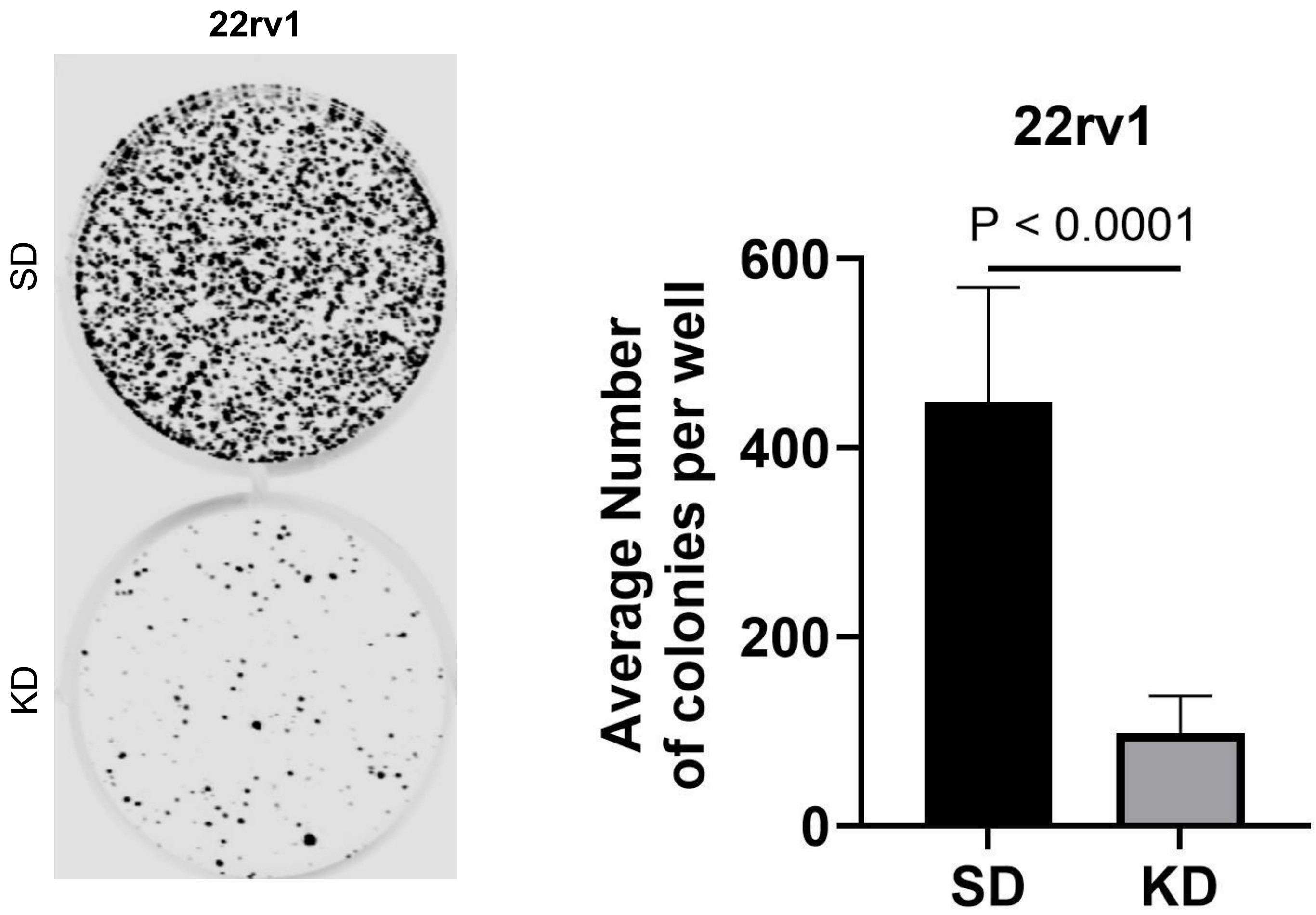

Figure S2. (A,B) Protein expression levels of CYR61 were significantly increased in metastatic PCa cells

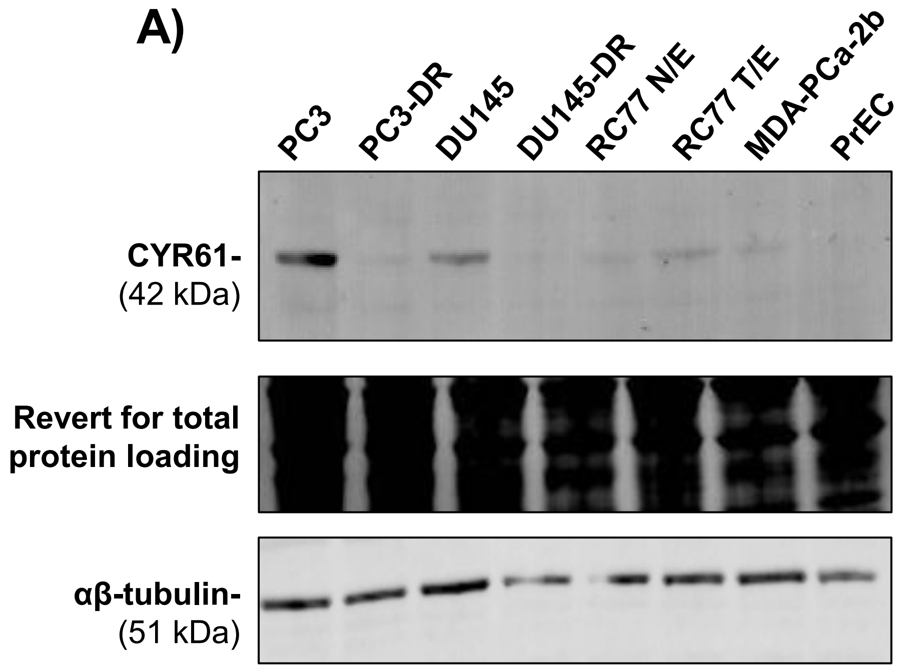

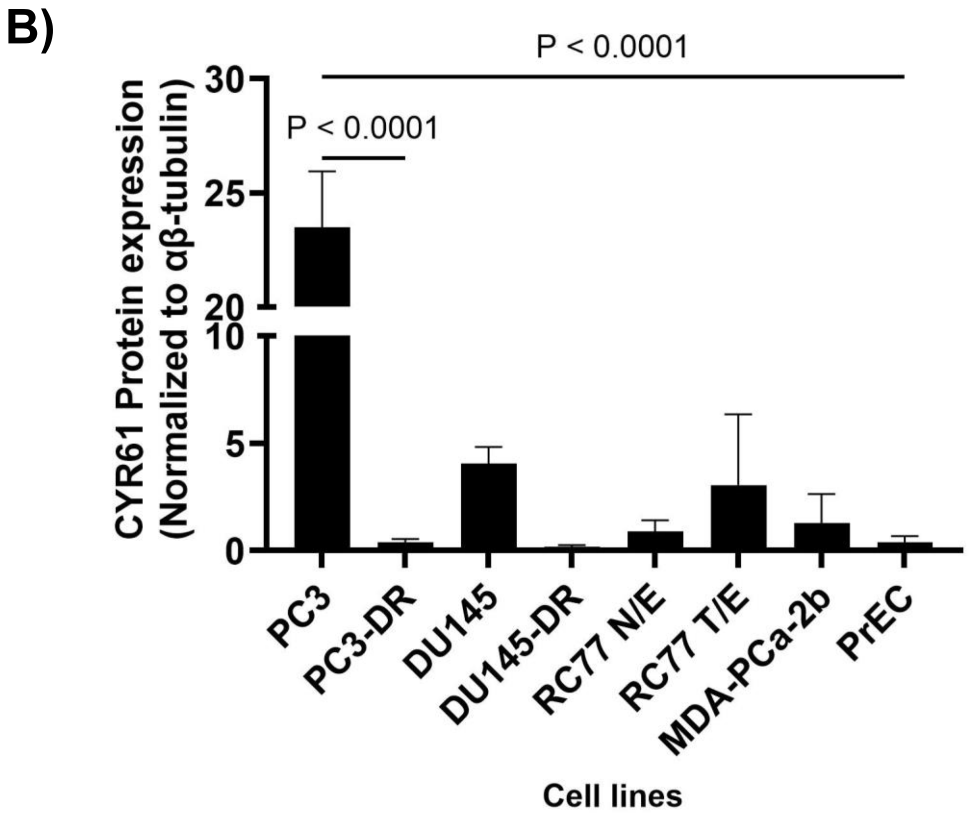

Figure S3. AR-V7 expression in PC3, high proliferative LNCaP and 22rv1 cells.

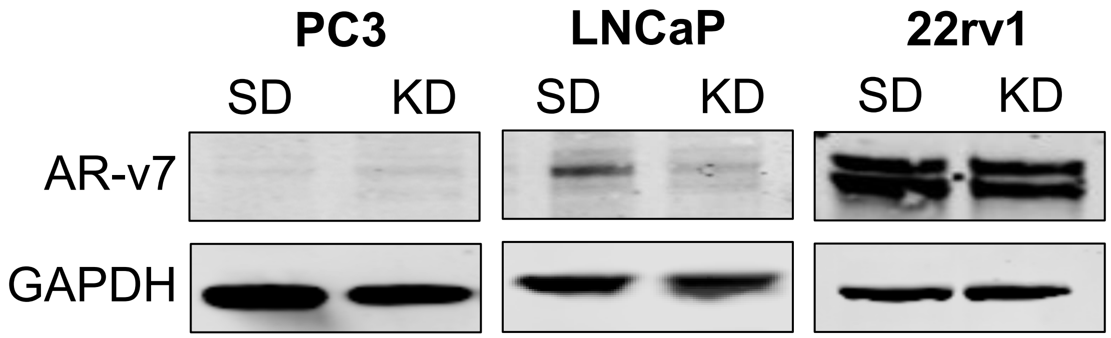

Figure S4. SF3B1 splicing factor expression validation in PC3, high proliferative LNCaP, and 22rv1 cells.

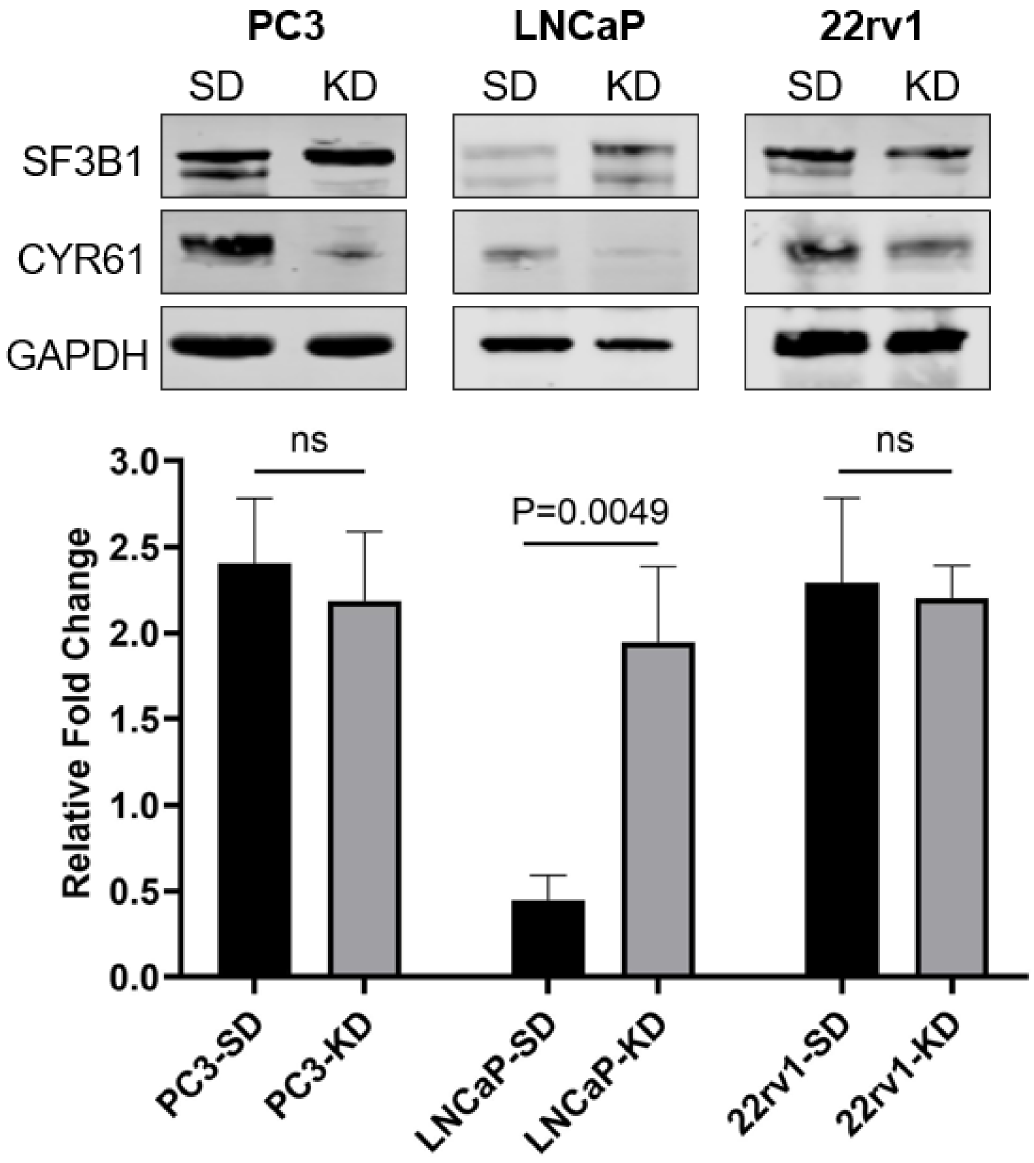

Supplement: Supplementary file 1 [file ijms-26-08991-s001.zip › ijms-3727461-supplementary.pdf]
